# Dapagliflozin and cognitive impairment: pharmacological mechanisms, translational evidence, and future directions

**DOI:** 10.3389/fphar.2026.1895764

**Published:** 2026-07-20

**Authors:** Ahmad H. Alhowail

**Affiliations:** Department of Pharmacology and Toxicology, College of Pharmacy, Qassim University, Buraydah, Saudi Arabia

**Keywords:** Alzheimer’s disease, cognitive impairment, dapagliflozin, mitochondrial dysfunction, neuroinflammation, neurovascular unit, oxidative stress, SGLT2 inhibitor

## Abstract

Cognitive impairment increasingly emerges at the intersection of type 2 diabetes mellitus, vascular brain injury, chronic kidney disease, heart failure, and neurodegeneration, prompting interest in therapies that modify shared metabolic and inflammatory drivers of brain vulnerability. Dapagliflozin, a sodium-glucose cotransporter 2 inhibitor widely used in type 2 diabetes and cardiorenal disease, has attracted attention as a candidate modulator of cognitive decline because its established peripheral pharmacology extends beyond glucose lowering to include natriuresis, blood pressure reduction, weight loss, improved insulin resistance, reduced oxidative and inflammatory stress, and favorable cardiorenal effects. In this review, we examine the pharmacological basis by which these systemic actions could influence the neurovascular unit, mitochondrial homeostasis, glial activation, autophagy-related signaling, and synaptic plasticity pathways implicated in cognitive impairment. We summarize preclinical evidence suggesting that dapagliflozin can improve cognitive performance and modulate pathways such as AMPK-mTOR, Wnt/β-catenin, CREB/BDNF, oxidative stress responses, and neuroinflammatory signaling in experimental models, while also critically evaluating the limitations of these models. We then assess the current human evidence, distinguishing observational studies that suggest lower dementia risk from randomized clinical evidence that has not yet established a definitive cognition-related benefit. We argue that dapagliflozin should currently be viewed not as a proven cognitive therapeutic, but as a mechanistically plausible metabolic-neurovascular intervention whose relevance may be greatest in metabolically vulnerable phenotypes. Finally, we outline key translational challenges, including uncertainty regarding direct central target engagement, the need for biomarker-enriched trial designs, and the importance of integrating pharmacological, vascular, and neurodegenerative frameworks in future studies.

## Graphical Abstract

**Figure:**
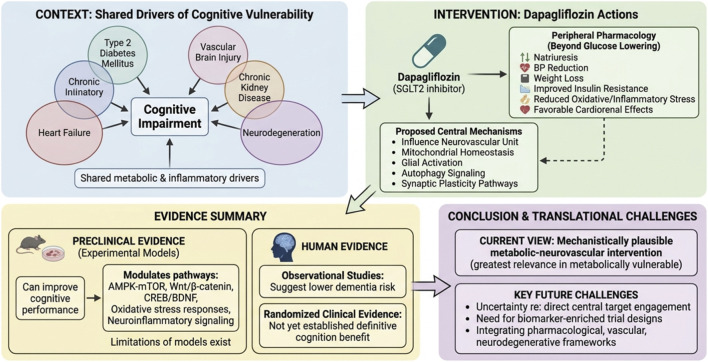

## 1 Introduction

Cognitive impairment increasingly develops at the convergence of metabolic disease, vascular brain injury, chronic kidney disease, heart failure, and neurodegeneration (Livingston et al., 2024; Biessels and Despa, 2018; van Sloten et al., 2020; Craft, 2009). This overlap is particularly evident in individuals with type 2 diabetes mellitus, in whom hyperglycemia, insulin resistance, endothelial dysfunction, oxidative stress, chronic low-grade inflammation, and microvascular injury collectively increase susceptibility to cognitive decline and dementia (Biessels and Despa, 2018; Arnold et al., 2018; van Sloten et al., 2020). These interacting processes support a shift away from viewing cognitive impairment as an exclusively neurodegenerative disorder and toward a broader pathobiological framework in which systemic cardiometabolic stress contributes to brain vulnerability through vascular, inflammatory, and bioenergetic mechanisms (Livingston et al., 2024; Wardlaw et al., 2019; Sweeney et al., 2018; Zlokovic, 2011).

The clinical rationale for dapagliflozin is fundamentally anchored in its ability to modify these intersecting systemic vulnerabilities across multiple disease states. Initially indicated solely for glycemic control in type 2 diabetes, where it reduces glucotoxicity, insulin resistance, and the downstream oxidative and inflammatory cascades that accelerate cerebral microvascular injury, its therapeutic application has expanded considerably. In heart failure, dapagliflozin reduces volume overload, improves cardiac output, and attenuates the chronic cerebral hypoperfusion that is increasingly recognized as a driver of cognitive decline in patients with reduced ejection fraction (McMurray et al., 2019; Solomon et al., 2022). In chronic kidney disease, it mitigates the accumulation of uremic toxins, vascular calcification, and systemic inflammatory activation that collectively impair blood-brain barrier integrity and accelerate neurovascular aging (Heerspink et al., 2020). This broad clinical utility positions dapagliflozin not merely as an antidiabetic agent, but as a comprehensive metabolic-neurovascular intervention whose relevance to cognitive health derives from its capacity to simultaneously address multiple upstream pathobiological drivers of brain vulnerability in highly comorbid populations.

Within this context, dapagliflozin has emerged as a pharmacologically plausible candidate for modulating pathways relevant to cognitive impairment (Ferrannini, 2017; Wiviott et al., 2019; McMurray et al., 2019; Heerspink et al., 2020). Dapagliflozin is a selective sodium-glucose cotransporter 2 inhibitor with established efficacy in type 2 diabetes, heart failure, and chronic kidney disease (Heerspink et al., 2020). Although its primary site of action is the renal proximal tubule, its biological effects extend well beyond glycemic control and include natriuresis, modest weight reduction, blood pressure lowering, improvement in insulin resistance, attenuation of oxidative and inflammatory stress, and favorable cardiorenal hemodynamic effects (Ferrannini, 2017; Wiviott et al., 2019; Vallon and Thomson, 2017). These systemic actions have generated interest in whether dapagliflozin might influence the neurovascular unit, cerebral perfusion, mitochondrial homeostasis, glial activation, and other mechanistic domains implicated in cognitive decline (Wardlaw et al., 2019; Mosconi, 2005; Sweeney et al., 2018; Heneka et al., 2015; Swerdlow, 2018).

However, the rationale for dapagliflozin in cognitive impairment requires careful evidentiary framing. Preclinical studies suggest that SGLT2 inhibition may improve behavioral outcomes and modulate molecular pathways linked to neuroinflammation, oxidative injury, mitochondrial dysfunction, autophagy-related signaling, and synaptic plasticity (El-Safty et al., 2022; Samman et al., 2023; Kostrzewska et al., 2025). By contrast, human evidence remains less definitive. Observational studies have reported associations between SGLT2 inhibitor exposure and lower dementia incidence, but such findings remain vulnerable to residual confounding, competing-risk effects, and treatment-selection bias (Pan et al., 2025; Shin et al., 2024). Randomized trials, moreover, were not primarily designed to evaluate cognition and have not yet established a definitive class-level or dapagliflozin-specific benefit for preventing cognitive decline (Burns et al., 2025; Seminer et al., 2025). Accordingly, dapagliflozin should not be regarded as a proven cognitive therapeutic, but rather as a mechanistically promising intervention whose relevance may be greatest in metabolically and vascularly vulnerable populations.

For a pharmacology-focused review, the central question is therefore not simply whether dapagliflozin is associated with better cognitive outcomes, but how its established peripheral pharmacology might intersect with biological pathways that influence brain resilience. This perspective is especially important for Frontiers in Pharmacology, which prioritizes mechanistic insight into drug action rather than purely clinical or statistical description. Framing dapagliflozin as a potential metabolic-neurovascular intervention allows a more rigorous analysis of how systemic metabolic unloading, vascular protection, inflammatory modulation, and altered cellular energetics may converge on processes relevant to cognitive impairment (Wardlaw et al., 2019; Mosconi, 2005; Sweeney et al., 2018; Heneka et al., 2015; Swerdlow, 2018; Vallon and Thomson, 2017).

In this review, we examine the pharmacological basis for the potential cognitive relevance of dapagliflozin across four interconnected levels: its established systemic actions; the pathophysiological links between cardiometabolic disease and brain injury; the preclinical evidence supporting neuroprotective mechanisms; and the emerging but still inconclusive clinical and translational data. We further address major controversies, including the relative importance of peripheral versus direct central mechanisms, the limitations of currently available animal and human studies, and the key design features required for future biomarker-enriched trials. By integrating metabolic, vascular, inflammatory, and neurodegenerative frameworks, this review aims to define the current state of the field and clarify where dapagliflozin may ultimately fit within the translational pharmacology of cognitive impairment.

## 2 Pharmacology of dapagliflozin relevant to cognition

Dapagliflozin is a highly selective inhibitor of SGLT2 expressed predominantly in the proximal renal tubule, where it reduces glucose and sodium reabsorption and thereby promotes glycosuria and natriuresis (Ferrannini, 2017; Vallon and Thomson, 2017). Although developed as an antidiabetic agent, its therapeutic profile now extends to heart failure and chronic kidney disease, underscoring the breadth of its systemic physiological effects (Wiviott et al., 2019; McMurray et al., 2019; Heerspink et al., 2020). From the perspective of cognitive impairment, the relevance of dapagliflozin lies less in direct symptomatic modulation of cognition and more in its capacity to modify upstream metabolic and vascular stressors that contribute to brain injury (Biessels and Despa, 2018; Wardlaw et al., 2019; van Sloten et al., 2020).

Several established actions of dapagliflozin may be mechanistically relevant to brain health. By improving glycemic control and reducing glucotoxicity, the drug may attenuate downstream molecular processes linked to oxidative stress, endothelial dysfunction, and inflammatory activation (Ferrannini, 2017; Arnold et al., 2018; De Felice and Ferreira, 2014). Its blood pressure lowering and natriuretic effects may also lessen arterial stiffness, microvascular injury, and cerebral hypoperfusion, all of which are implicated in vascular contributions to cognitive impairment (Wardlaw et al., 2019; van Sloten et al., 2020; Sweeney et al., 2018; Zlokovic, 2011). In parallel, modest improvements in weight, insulin resistance, and cardiorenal hemodynamics may reduce the chronic multisystem burden that amplifies neurovascular vulnerability in patients with diabetes, chronic kidney disease, and heart failure (Wiviott et al., 2019; McMurray et al., 2019; Heerspink et al., 2020; Zelniker et al., 2019; Vaduganathan et al., 2022; Vallon and Thomson, 2017).

A more speculative but increasingly discussed aspect of dapagliflozin pharmacology relates to altered substrate handling and cellular energetics. SGLT2 inhibition may promote mild ketogenesis and shifts in whole-body energy metabolism, raising the possibility that it could indirectly support cerebral bioenergetics under conditions of metabolic stress (Mosconi, 2005; Vallon and Thomson, 2017). Similarly, systemic unloading may affect redox balance, inflammatory tone, and mitochondrial homeostasis, which in turn could influence neuronal and glial resilience (Pawlos et al., 2021; Kostrzewska et al., 2025; Heneka et al., 2015; Swerdlow, 2018). These effects remain mechanistically attractive, but they do not by themselves establish direct central nervous system activity.

**FIGURE 1:**
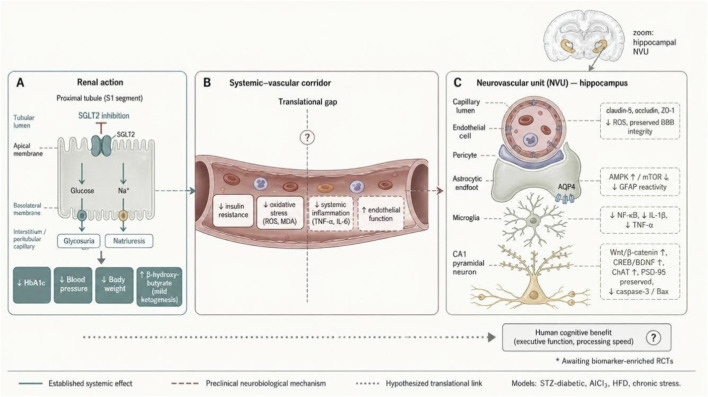
Dapagliflozin modulates cognition via a renal-systemic neurovascular axis. **(A)** SGLT2 inhibition at the renal proximal tubule produces glycosuria and natriuresis, driving established systemic effects (↓HbA1c, ↓blood pressure, ↓body weight, mild ketogenesis). **(B)** Systemic-vascular corridor carrying reduced insulin resistance, oxidative stress (ROS, MDA), and pro-inflammatory tone (TNF-α, IL-6) toward the cerebral circulation; a vertical boundary marks the translational gap between established peripheral effects and inferred central mechanisms. **(C)** Hippocampal neurovascular unit showing the proposed cell-specific actions: preserved BBB tight-junction integrity (claudin-5, occludin, ZO-1), reduced astrocytic reactivity with AMPK/mTOR rebalancing, attenuated microglial NF-κB signaling, and neuronal Wnt/β-catenin, CREB/BDNF, and cholinergic preservation with reduced apoptotic markers. Color saturation encodes evidence tier: solid/saturated, established systemic effect; dashed/desaturated, preclinical mechanism (STZ-diabetic, AlCl_3_, HFD, and chronic-stress models); dotted, hypothesized translational link to human cognitive benefit, awaiting biomarker-enriched RCTs. Abbreviations: BBB, blood–brain barrier; ROS, reactive oxygen species; MDA, malondialdehyde; RCTs, randomized controlled trials.

Whether dapagliflozin exerts clinically meaningful direct effects within the brain remains unresolved. Although SGLT2-related pathways and off-target metabolic signaling have been discussed in experimental work, evidence for substantial central target engagement in humans remains limited (Kostrzewska et al., 2025; Vallon and Thomson, 2017). This distinction is important, because the translational value of dapagliflozin may depend predominantly on peripheral disease modification rather than direct neuronal pharmacology. Accordingly, the most defensible current framework is to view dapagliflozin as a systemic metabolic-neurovascular modulator whose putative relevance to cognition arises from the remodeling of whole-body disease biology rather than from confirmed primary brain penetration. Figure 1 provides a visual summary of these proposed mechanistic pathways linking dapagliflozin’s systemic pharmacology to potential cognitive protection.

## 3 Pathophysiological links between cardiometabolic disease and cognitive impairment

The relationship between cardiometabolic disease and cognitive impairment is multidimensional and extends beyond traditional glycemic paradigms (Livingston et al., 2024; Biessels and Despa, 2018; Arnold et al., 2018; Craft, 2009). In type 2 diabetes and related disorders, chronic hyperglycemia, insulin resistance, dyslipidemia, endothelial dysfunction, and low-grade inflammation act in concert to damage both large and small vessels, impair metabolic signaling, and reduce tissue resilience (Biessels and Despa, 2018; Arnold et al., 2018; van Sloten et al., 2020; De Felice and Ferreira, 2014). The brain is particularly vulnerable to these effects because it depends on tightly regulated substrate delivery, vascular integrity, and mitochondrial function (Mosconi, 2005; Sweeney et al., 2018; Swerdlow, 2018; Zlokovic, 2011). Repeated exposure to systemic metabolic stress can therefore accelerate the development of white matter injury, blood-brain barrier dysfunction, impaired neurovascular coupling, and progressive decline in synaptic and neuronal integrity (Wardlaw et al., 2019; van Sloten et al., 2020; Sweeney et al., 2018; Zlokovic, 2011).

Vascular mechanisms are central to this process. Hypertension, arterial stiffness, endothelial activation, and chronic renal dysfunction contribute to cerebral small-vessel disease, altered perfusion, and impaired clearance of metabolites and toxic proteins (Wardlaw et al., 2019; Sweeney et al., 2018; Zlokovic, 2011). These changes may not only promote vascular cognitive impairment directly (Dichgans and Leys, 2017) but also amplify the clinical expression of neurodegenerative pathology (Livingston et al., 2024; Sweeney et al., 2018; Craft, 2009). Thus, the metabolic and vascular environments in which dementia develops are often inseparable from one another, particularly in older adults with multiple cardiometabolic comorbidities (Livingston et al., 2024; Craft, 2009).

A critical and often underappreciated observation is that the primary clinical indications for SGLT2 inhibitor therapy, type 2 diabetes, hypertension, heart failure, and chronic kidney disease, are themselves established risk factors for neurodegenerative diseases. Diabetes and vascular dysfunction do not merely coexist with neurodegeneration; they actively accelerate it through shared pathogenic mechanisms including endothelial injury, chronic hypoperfusion, and neuroinflammation. The significance of vascular pathogenesis extends even beyond classical dementias to atypical parkinsonian disorders, as demonstrated by Dunalska et al. (2021), who showed that cerebral hypoperfusion and vascular lesions contribute significantly to the clinical manifestation of corticobasal syndrome. This underscores the broad relevance of vascular health to diverse forms of neurodegeneration and supports the rationale for targeting these shared upstream mechanisms. The relevance of dapagliflozin to cognitive deterioration is also likely domain-specific. Cognitive domains most dependent on intact subcortical-frontal networks and adequate cerebral perfusion, such as executive function, processing speed, and complex attention, are classically impaired in vascular cognitive impairment and diabetes-related cognitive decline (Dichgans and Leys, 2017; Biessels and Despa, 2018). These domains may be most responsive to interventions that improve vascular and metabolic health. By contrast, primary episodic memory deficits, which characterize early Alzheimer’s disease and reflect hippocampal and medial temporal lobe neurodegeneration, may be less directly amenable to systemic metabolic modification alone.

Inflammatory and oxidative mechanisms add a further layer of convergence. Chronic metabolic disease is associated with increased reactive oxygen species, redox imbalance, activation of inflammatory signaling pathways, and dysregulated glial responses (Heneka et al., 2015; Mattson and Arumugam, 2018; Swerdlow, 2018; De Felice and Ferreira, 2014). Over time, these disturbances may disrupt mitochondrial function, impair autophagic flux, and compromise synaptic plasticity (Mosconi, 2005; Heneka et al., 2015; Swerdlow, 2018). Such processes are relevant not only to diabetes-related cognitive decline but also to broader neurodegenerative cascades, including those associated with Alzheimer-type pathology (Livingston et al., 2024; Heneka et al., 2015; Swerdlow, 2018; De Felice and Ferreira, 2014). For this reason, interventions that reduce systemic metabolic injury may have indirect but biologically meaningful effects on the brain even if they are not classical neuropharmacological agents.

This integrated framework provides the biological rationale for studying dapagliflozin in cognitive impairment. If a therapy can reduce glucotoxicity, improve vascular function, attenuate inflammatory stress, and support mitochondrial or energetic stability, then it may help modify the conditions under which cognitive decline progresses (Wiviott et al., 2019; McMurray et al., 2019; Heerspink et al., 2020; Vallon and Thomson, 2017). The key translational challenge is to determine whether such upstream systemic benefits are sufficient to generate measurable and clinically relevant effects on cognition in humans.

## 4 Candidate mechanisms by which dapagliflozin may influence cognitive trajectories

A mechanistic framework linking dapagliflozin to cognition must begin with its established systemic effects and then proceed cautiously toward brain-relevant pathways. The strongest evidence supports the idea that dapagliflozin reduces metabolic and hemodynamic stress through glycosuria, natriuresis, blood pressure reduction, improved insulin sensitivity, and favorable cardiorenal effects (Wiviott et al., 2019; McMurray et al., 2019; Heerspink et al., 2020; Vallon and Thomson, 2017). These changes may lower the chronic burden of endothelial injury, oxidative stress, and inflammatory signaling that contributes to neurovascular dysfunction (Wardlaw et al., 2019; Sweeney et al., 2018; Heneka et al., 2015; De Felice and Ferreira, 2014).

One important pathway involves the neurovascular unit, which comprises endothelial cells, pericytes, astrocytes, and neurons and is essential for maintaining blood-brain barrier integrity and cerebral perfusion (Wardlaw et al., 2019; Sweeney et al., 2018; Zlokovic, 2011). By reducing systemic vascular stress and improving endothelial function, dapagliflozin may help preserve neurovascular coupling and reduce microvascular injury (Wardlaw et al., 2019; van Sloten et al., 2020; Sweeney et al., 2018; Zlokovic, 2011). This mechanism is particularly relevant in patients with diabetes, hypertension, and chronic kidney disease, where neurovascular dysfunction may be a major determinant of cognitive decline (Biessels and Despa, 2018; Wardlaw et al., 2019; van Sloten et al., 2020).

A second mechanistic domain concerns mitochondrial energetics and oxidative stress. Experimental data suggest that dapagliflozin may improve redox balance, reduce lipid peroxidation, and support mitochondrial efficiency under conditions of metabolic overload (El-Safty et al., 2022; Samman et al., 2023; Swerdlow, 2018). These effects are biologically attractive because impaired bioenergetics and oxidative injury contribute to synaptic dysfunction and neuronal vulnerability across a wide range of cognitive disorders (Mosconi, 2005; Swerdlow, 2018; De Felice and Ferreira, 2014). Closely related to this is the possibility that SGLT2 inhibition may influence cellular stress-response pathways such as AMPK-mTOR signaling and autophagic regulation, thereby affecting proteostasis and survival signaling in vulnerable neural tissue (Samman et al., 2023; Kostrzewska et al., 2025).

Neuroinflammation represents another key target. Preclinical studies suggest that dapagliflozin can reduce inflammatory signaling and glial reactivity, including pathways relevant to astrocytic and microglial activation (El-Safty et al., 2022; Samman et al., 2023; Kostrzewska et al., 2025; Heneka et al., 2015). If confirmed in translational settings, such effects would support a model in which systemic metabolic improvement secondarily dampens neuroimmune activation (Heneka et al., 2015; De Felice and Ferreira, 2014). In parallel, reported changes in Wnt/β-catenin, CREB, and BDNF-related signaling raise the possibility that dapagliflozin may influence synaptic plasticity and memory-related circuitry (El-Safty et al., 2022; Samman et al., 2023). These data remain predominantly experimental, but they offer a plausible mechanistic bridge between peripheral metabolic pharmacology and cognitive phenotypes (El-Safty et al., 2022; Samman et al., 2023; Kostrzewska et al., 2025).

When evaluating these candidate mechanisms, it is essential to establish a clear hierarchy of evidentiary support. The most robust evidence supports dapagliflozin’s ability to reduce systemic vascular stress, improve endothelial function, and attenuate systemic inflammation, effects that are well-documented in large clinical trials and have clear mechanistic links to neurovascular protection (Wiviott et al., 2019; McMurray et al., 2019; Heerspink et al., 2020). Intermediate-level evidence supports modulation of mitochondrial energetics and oxidative stress, based on consistent preclinical findings across multiple models (El-Safty et al., 2022; Samman et al., 2023). In contrast, claims regarding direct central modulation of Wnt/β-catenin, CREB/BDNF, or AMPK-mTOR pathways within the brain remain largely speculative, confined to experimental animal models using supraphysiological doses or acute injury paradigms, and require rigorous human validation before translational conclusions can be drawn. The relevance of dapagliflozin to specific neurodegenerative pathologies also requires careful differentiation. Its effects on core Alzheimer’s pathology, amyloid-beta aggregation and primary tauopathies, are currently tentative and likely indirect at best (Burns et al., 2025; Seminer et al., 2025). However, dapagliflozin may exert its most meaningful effects in the context of co-existing pathologies, which is the clinical reality for most patients with late-life cognitive impairment. In mixed dementia, where Alzheimer’s pathology co-occurs with significant cerebrovascular disease, systemic metabolic and vascular unloading could reduce the cumulative neuroinflammatory and oxidative burden that lowers the threshold for clinical expression of cognitive symptoms. Emerging preclinical evidence also suggests potential relevance to other neurodegenerative conditions; SGLT2 inhibitors have shown neuroprotective effects in experimental models of Parkinson’s disease, though clinical translation remains unexplored (Kostrzewska et al., 2025; Pawlos et al., 2021). By modifying these compounding systemic factors, dapagliflozin may delay the clinical threshold at which underlying neurodegeneration manifests as functional impairment, even if it does not reverse the primary proteinopathy itself.

## 5 Preclinical evidence

Preclinical studies provide much of the current mechanistic enthusiasm for dapagliflozin in cognitive impairment. Across diabetic and Alzheimer-like models, the drug has been associated with improved performance in behavioral paradigms relevant to memory and learning, including tasks dependent on hippocampal function (El-Safty et al., 2022; Samman et al., 2023; Kostrzewska et al., 2025). These findings support the possibility that SGLT2 inhibition can influence neural processes involved in cognition, at least in experimental settings characterized by metabolic or toxic injury. Table 1 summarizes representative preclinical studies examining dapagliflozin’s effects on cognition-relevant outcomes.

**TABLE 1:** Representative preclinical studies of dapagliflozin and cognition. Each row corresponds to a specific published study; findings are reported as stated in the original publication.

Study	Experimental model	Dapagliflozin regimen	Cognitive assay/Outcome	Main mechanistic findings	Key translational limitation
El-Safty et al. (2022)	Streptozotocin-induced diabetic rat model	Oral dapagliflozin; duration per original study	Morris water maze and Object Location memory task; hippocampal Wnt/β-catenin, CREB signaling, cholinergic markers, oxidative stress, apoptosis	Improved spatial memory; restored Wnt/β-catenin and CREB signaling; improved cholinergic function; reduced oxidative stress and apoptosis	STZ model reflects severe β-cell injury; limited representation of chronic, aged T2D with multimorbidity
Samman et al. (2023)	Aluminum-chloride-induced Alzheimer-like rat model	Dapagliflozin 1 and 5 mg/kg/day, 4 weeks	Morris water maze; Y-maze	Improved spatial memory; modulation of AMPK/mTOR signaling; improved glucose metabolism; reduced oxidative stress	Chemical AD-like model captures selected features only; does not reproduce full amyloid/tau biology
Abdelhafez et al. (2025)	Doxorubicin-induced cognitive impairment rat model	Dapagliflozin 2 mg/kg/day, oral, 28 days	Novel Object recognition test, Y-maze, Open field test; NOX4, MDA, SOD, GSH, IL-1β, TNF-α, NF-κB, AKT/GSK-3β, Wnt/β-catenin, caspase-3	Reduced neuroinflammation (TNF-α, IL-1β, NF-κB), oxidative stress (NOX4, MDA), and apoptosis (caspase-3); modulated AKT/GSK-3β and Wnt/β-catenin pathways; prevented doxorubicin-induced cognitive decline	Acute chemotoxicity model; distinct pathophysiology from age-related neurodegeneration
Sa-Nguanmoo et al. (2017)	High-fat diet-induced obese-insulin resistant rat model	Dapagliflozin 1 mg/kg/day, oral, 4 weeks (after 12 weeks HFD)	Morris water maze; brain mitochondrial function, insulin signaling, synaptic plasticity (LTP)	Prevented HFD-induced cognitive decline; improved brain mitochondrial function, insulin signaling, and hippocampal synaptic plasticity; reduced brain oxidative stress and apoptosis	Focuses primarily on systemic metabolic correction rather than direct central target engagement

Abbreviations: AMPK, AMP-activated protein kinase; CREB, cAMP, response element-binding protein; mTOR, mechanistic target of rapamycin; STZ, streptozotocin; T2D, type 2 diabetes. Preclinical data support mechanistic plausibility but do not establish human efficacy.

In models of diabetes-related cognitive impairment, dapagliflozin has been reported to improve behavioral outcomes while modulating signaling pathways linked to neuronal survival, synaptic plasticity, and oxidative stress (El-Safty et al., 2022; Kostrzewska et al., 2025). Such effects are often interpreted within a framework of reduced glucotoxicity, improved insulin signaling, and attenuation of inflammatory or apoptotic cascades (Arnold et al., 2018; El-Safty et al., 2022; De Felice and Ferreira, 2014). In Alzheimer-like models, reported benefits include modulation of AMPK-mTOR signaling, improvement in redox balance, and attenuation of injury-related molecular markers (Samman et al., 2023; Kostrzewska et al., 2025). These observations reinforce the notion that dapagliflozin may influence shared downstream pathways connecting metabolic stress to neuronal dysfunction.

A balanced appraisal of this preclinical literature requires explicit acknowledgment of both its strengths and limitations. Key strengths include the ability to isolate specific molecular pathways under controlled conditions, the demonstration of dose-response relationships, and the identification of convergent mechanisms (such as AMPK-mTOR modulation and oxidative stress reduction) across independent laboratories and different experimental paradigms. These features provide genuine mechanistic plausibility and help guide biomarker selection for future clinical studies. However, significant weaknesses limit the translational value of the current preclinical evidence. First, many studies rely on acute chemical induction models (e.g., high-dose streptozotocin or aluminum chloride), which produce rapid, severe injury that poorly mimics the chronic, low-grade, and multimorbid nature of human cognitive impairment. Second, most animal models fail to incorporate the co-existence of aging, frailty, polypharmacy, and mixed vascular-neurodegenerative pathology that characterizes the typical patient population in whom dapagliflozin would be considered. Third, conflicting data regarding the extent of SGLT2 expression in the mammalian brain create fundamental uncertainty about whether observed central effects result from direct target engagement or are entirely secondary to peripheral metabolic improvements. These gaps represent critical knowledge deficits that must be addressed before preclinical enthusiasm can be translated into clinical confidence.

These limitations do not diminish the importance of the preclinical literature, but they do define its proper role. At present, experimental studies provide mechanistic plausibility and biological direction rather than clinical proof (El-Safty et al., 2022; Samman et al., 2023; Kostrzewska et al., 2025). Their greatest value lies in identifying candidate pathways, guiding biomarker selection, and helping define which patient phenotypes may be most informative for translational investigation (El-Safty et al., 2022; Samman et al., 2023; Kostrzewska et al., 2025; Jack et al., 2018; Palmqvist et al., 2020; Hansson, 2021).

## 6 Clinical and translational evidence

The current human evidence for dapagliflozin and cognition is suggestive but inconclusive. Observational studies and cohort-based meta-analyses have reported associations between SGLT2 inhibitor use and reduced dementia incidence in patients with type 2 diabetes, generating substantial interest in the possibility of class-level neuroprotection (Pan et al., 2025; Shin et al., 2024; Song et al., 2025; Wu et al., 2025; Zhou et al., 2020). These findings are clinically important because they align with the broader hypothesis that improvement in systemic metabolic and vascular health may influence long-term cognitive outcomes (Livingston et al., 2024; Biessels and Despa, 2018; van Sloten et al., 2020). Table 2 provides an overview of the current clinical evidence linking SGLT2 inhibitors to cognitive outcomes.

**TABLE 2:** Clinical evidence for SGLT2 inhibitors and cognitive outcomes. Each row corresponds to a specific published study or formal meta-analysis; findings are reported as stated in the original publication.

Study/Evidence item	Study type	Population	Exposure/Comparator	Endpoint	Main finding	Key limitation
Pan et al. (2025)	Meta-analysis of cohort studies	∼331,908 patients with T2D	SGLT2i vs. DPP-4i, GLP-1 RA, insulin, or other antidiabetic regimens	Incident dementia	Pooled risk ratio ≈0.77; lower dementia risk in real-world populations	Residual confounding; comparator heterogeneity; administrative-data misclassification; healthy-user bias
Shin et al. (2024)	Comparative observational study	Patients with T2D	SGLT2i vs. comparator antidiabetic agents	Dementia or cognitive-risk outcome	SGLT2i users showed significantly lower risk of dementia compared to DPP-4i users (adjusted HR 0.65, 95% CI 0.54–0.78)	Non-randomized; confounding by indication and treatment selection
Song et al. (2025)	Systematic review and meta-analysis	13 observational studies; patients with T2D	SGLT2i vs. incretin mimetics or other antidiabetics	Overall dementia risk	SGLT2i significantly reduced dementia risk (HR 0.82, 95% CI: 0.73–0.91); empagliflozin most consistently protective	Relies on observational data; potential healthy-user bias; no dapagliflozin-specific subgroup
Seminer et al. (2025)	Systematic review/meta-analysis of RCTs	∼164,531 participants across 26 trials in diabetes, HF, or CKD	Cardioprotective glucose-lowering therapies (SGLT2is, GLP-1RAs, pioglitazone) vs. controls	Cognitive impairment or dementia, often as secondary/adverse-event outcomes	No overall significant reduction in dementia (OR 0.83, 95% CI 0.60–1.14); GLP-1RAs showed significant reduction (OR 0.55); SGLT2is did not (OR 1.20, 95% CI 0.67–2.17)	Trials not designed for cognition; limited follow-up; low event rates; relatively young populations
Burns et al. (2025)	Randomized controlled trial	Participants with early Alzheimer’s disease	Dapagliflozin 10 mg vs. placebo	Feasibility, safety, mechanistic brain-related outcomes	No significant change in primary outcome (cerebral N-acetylaspartate); feasible and safe; secondary metabolic effects (reduced HbA1c, fat mass) and exploratory cognitive signal (improved stroop interference)	Pilot scale; short duration; not designed for firm efficacy conclusions

Abbreviations: CKD, chronic kidney disease; DPP-4, dipeptidyl peptidase-4; GLP-1, RA, glucagon-like peptide-1, receptor agonist; HF, heart failure; MCI, mild cognitive impairment; RCT, randomized controlled trial; SGLT2i, sodium-glucose cotransporter two inhibitor; T2D, type 2 diabetes. Observational evidence is supportive but causal inference is limited; randomized cognition-specific evidence remains insufficient.

Nevertheless, observational data require careful interpretation. Dementia outcomes are highly susceptible to confounding by indication, healthy-user bias, competing mortality, diagnostic misclassification, and differences in healthcare utilization (Pan et al., 2025; Shin et al., 2024). Patients prescribed SGLT2 inhibitors may differ systematically from comparator groups in ways that are not fully captured by statistical adjustment. As a result, even strong associations cannot be assumed to reflect causal drug effects on cognition.

Several alternative explanations for these observational findings must be critically considered. The apparent reduction in dementia risk among SGLT2 inhibitor users might partially reflect better overall cardiovascular care, improved medication adherence, avoidance of severe hypoglycemic episodes (which are independently neurotoxic), or a healthy-user effect whereby patients prescribed newer agents are systematically healthier than those on older therapies. These confounders are difficult to fully eliminate even with propensity-score matching or active-comparator designs. Furthermore, a striking discrepancy exists between observational and randomized evidence that demands explicit discussion. While cohort studies consistently report 20%–50% reductions in dementia incidence with SGLT2 inhibitor use (Pan et al., 2025; Shin et al., 2024), the only dapagliflozin-specific randomized controlled trial in early Alzheimer’s disease (Burns et al., 2025) found no significant improvement in cognitive test performance or AD-specific biomarkers over its study period. This conflict likely reflects differences in population selection (metabolically healthy AD patients vs. diabetic cohorts), study duration (months vs. years of follow-up), endpoint sensitivity (clinical cognitive scales vs. dementia diagnosis), and the fundamental distinction between preventing metabolic-vascular contributions to cognitive decline *versus* reversing established neurodegeneration. It is also necessary to distinguish between potential class effects of SGLT2 inhibitors and dapagliflozin-specific properties. The majority of observational data suggesting reduced dementia incidence evaluates the SGLT2 inhibitor class as a whole. A recent systematic review and meta-analysis by Song et al. (2025) found that SGLT2 inhibitors significantly reduced overall dementia risk compared to incretin mimetics (HR 0.82, 95% CI: 0.73–0.91), with empagliflozin emerging as the most consistently protective individual agent. Preclinically, both empagliflozin and dapagliflozin demonstrate comparable neuroprotective effects against amyloid-beta-induced neurotoxicity *in vitro*. Given the shared pharmacological mechanisms of natriuresis, glycemic control, and vascular unloading across the class, it is highly probable that the observed cognitive benefits represent predominantly a class effect, though definitive head-to-head clinical trials comparing individual SGLT2 inhibitors for cognitive endpoints have not been conducted.

Randomized evidence has not yet provided definitive confirmation of a cognition-related benefit. Most trials involving dapagliflozin or the broader SGLT2 inhibitor class were designed around cardiovascular, renal, or glycemic endpoints rather than neurocognitive outcomes (Wiviott et al., 2019; McMurray et al., 2019; Heerspink et al., 2020; Anker et al., 2021; Solomon et al., 2022; Perkovic et al., 2019; Neuen et al., 2019; Seminer et al., 2025). They often included relatively young populations, short follow-up periods, low event rates for dementia, and limited or absent cognitive phenotyping (Seminer et al., 2025). Under such conditions, failure to detect benefit does not necessarily refute the hypothesis, but it does mean that clinical efficacy remains unproven.

Early dapagliflozin-specific clinical exploration has suggested feasibility and acceptable safety in cognitively relevant populations, but these signals fall short of establishing therapeutic efficacy (Burns et al., 2025). Thus, the current state of the field is best described as a translational gap: preclinical data and observational studies support biological plausibility, whereas randomized clinical proof remains insufficient (Burns et al., 2025; El-Safty et al., 2022; Samman et al., 2023; Kostrzewska et al., 2025; Pan et al., 2025; Seminer et al., 2025; Shin et al., 2024). Bridging this gap will require studies explicitly designed to test cognition-related hypotheses rather than secondary extrapolation from non-neurological trials.

## 7 Patient phenotypes most likely to benefit

If dapagliflozin has clinically meaningful cognitive relevance, it is unlikely to be equally effective across all forms of impairment. A precision-pharmacology perspective suggests that benefit would be most plausible in individuals whose cognitive vulnerability is strongly shaped by metabolic and vascular disease (Livingston et al., 2024; Biessels and Despa, 2018; van Sloten et al., 2020; Craft, 2009). This includes patients with type 2 diabetes, insulin resistance, obesity, chronic kidney disease, heart failure, hypertension, and imaging or biomarker evidence of cerebral small-vessel disease or neurovascular dysfunction (Wiviott et al., 2019; McMurray et al., 2019; Heerspink et al., 2020; Wardlaw et al., 2019; van Sloten et al., 2020; Zlokovic, 2011).

Such enrichment is important for both mechanistic and methodological reasons. From a biological perspective, these populations are the ones in whom dapagliflozin’s established pharmacology is most likely to intersect with drivers of brain injury (Ferrannini, 2017; Wiviott et al., 2019; McMurray et al., 2019; Heerspink et al., 2020; Vallon and Thomson, 2017). From a trial-design perspective, restricting enrollment to metabolically vulnerable phenotypes may improve the likelihood of detecting a true effect by reducing heterogeneity and increasing mechanistic alignment between the drug and the target pathology (Jack et al., 2018; Palmqvist et al., 2020; Hansson, 2021; Zlokovic, 2011).

This framework also implies that dapagliflozin may be less relevant in individuals with advanced neurodegeneration in the absence of meaningful metabolic or vascular burden. In such settings, peripheral disease modification alone may be insufficient to alter cognitive trajectories (Burns et al., 2025; Seminer et al., 2025). Thus, future studies should move beyond broad diagnostic labels and instead define target populations according to integrated metabolic, vascular, renal, and neurodegenerative profiles (Jack et al., 2018; Palmqvist et al., 2020; Hansson, 2021).

## 8 Safety in cognitively impaired and older adults

Any proposal to reposition dapagliflozin in cognitively vulnerable populations must consider safety carefully. Although the drug is generally well tolerated in its approved indications, older adults with frailty, polypharmacy, impaired self-management, or fluctuating oral intake may face distinct risks (Wiviott et al., 2019; McMurray et al., 2019; Heerspink et al., 2020). Volume depletion, hypotension, genitourinary infections, and challenges in medication adherence may be particularly relevant in patients with established cognitive impairment (Wiviott et al., 2019; McMurray et al., 2019; Heerspink et al., 2020; Burns et al., 2025).

These considerations do not preclude translational investigation, but they reinforce the need for rigorous patient selection and monitoring. Future trials should incorporate careful assessment of renal function, hydration status, falls risk, functional capacity, and caregiver support (Burns et al., 2025). Safety monitoring should be integrated with mechanistic endpoints rather than treated as a peripheral concern, especially in populations where vulnerability to adverse events may influence both trial retention and clinical interpretability.

## 9 Future directions and trial roadmap

The next stage of research should move from broad association toward mechanism-informed clinical testing. The most informative future studies are unlikely to be unselected dementia-prevention trials. Instead, they should focus on biomarker-enriched, phenotype-specific populations in whom dapagliflozin’s known pharmacology is biologically most likely to intersect with brain injury pathways (Burns et al., 2025; Jack et al., 2018; Palmqvist et al., 2020; Hansson, 2021; Zlokovic, 2011). Such trials should be adequately powered, sufficiently long in duration, and prospectively designed around cognitive and mechanistic endpoints (Burns et al., 2025; Seminer et al., 2025).

A rational Phase IIb design would include individuals with metabolic-vascular risk enrichment, such as type 2 diabetes with early cognitive decline, chronic kidney disease with neurovascular burden, or multimorbid patients with vascular and inflammatory risk profiles (Livingston et al., 2024; Biessels and Despa, 2018; Heerspink et al., 2020; Wardlaw et al., 2019; van Sloten et al., 2020). Primary and secondary endpoints should extend beyond conventional cognition scales to include mechanistic readouts such as structural MRI, cerebral perfusion imaging, white matter injury markers, and fluid biomarkers reflecting neurodegeneration and glial activation (Wardlaw et al., 2019; Sweeney et al., 2018; Jack et al., 2018; Hansson, 2021; Zlokovic, 2011). Candidate blood-based markers include p-tau217, p-tau181, neurofilament light chain, and GFAP, alongside systemic metabolic and inflammatory measures such as HbA1c, HOMA-IR, hsCRP, and related profiles (Jack et al., 2018; Palmqvist et al., 2020; Hansson, 2021).

Such an approach would help address one of the field’s central uncertainties: whether any cognitive signal observed with dapagliflozin reflects symptomatic improvement, slowed injury progression, or merely better overall systemic health (Burns et al., 2025; Seminer et al., 2025). By integrating neuroimaging, plasma biomarkers, and phenotypic enrichment, future studies can begin to separate peripheral from central mechanisms and define the patient groups most likely to derive measurable benefit (Jack et al., 2018; Palmqvist et al., 2020; Hansson, 2021; Zlokovic, 2011). Table 3 outlines the proposed biomarker framework for future translational trials.

**TABLE 3:** Proposed biomarker framework for future translational trials of dapagliflozin in cognitive impairment.

Biomarker domain	Candidate measure(s)	Mechanistic rationale	Translational relevance
Metabolic phenotype	HbA1c, fasting glucose, fasting insulin, HOMA-IR, β-hydroxybutyrate, lipid profile, body weight	Defines metabolic burden and pharmacodynamic response; tests the metabolic-unloading hypothesis	Identifies participants most likely to benefit; tracks whether cognitive signals follow systemic metabolic improvement
Cardio-renal status	eGFR, urine albumin-to-creatinine ratio, NT-proBNP, blood pressure, diuretic exposure	Quantifies vascular, renal, and congestion-related stress contributing to neurovascular injury	Connects established cardiorenal pharmacology to downstream brain outcomes
Neurodegeneration	Plasma neurofilament light chain (NfL); structural MRI (hippocampal volume, cortical thickness)	Captures neuronal injury and structural progression	Tests whether dapagliflozin modifies the tempo of neurodegenerative change
Alzheimer biology	Plasma p-tau217, p-tau181; amyloid PET; tau PET; CSF biomarkers (subset)	Stratifies treatment response by amyloid/tau burden	Distinguishes effects in pure AD biology from mixed metabolic-vascular phenotypes
Astroglial/Inflammatory state	GFAP, hsCRP, IL-6, TNF-related signatures	Tests whether reduced systemic inflammation and glial activation mediate benefit	Links peripheral inflammatory unloading to CNS-relevant biology
Neurovascular injury	White matter hyperintensity volume, lacunes, microbleeds, arterial spin labeling perfusion	Quantifies vascular cognitive impairment mechanisms, BBB-related injury, cerebral perfusion	Relevant for T2D-, CKD-, HF-, and small-vessel-disease-enriched populations
Brain metabolism	FDG-PET; magnetic resonance spectroscopy (NAA, lactate)	Evaluates cerebral substrate utilization, mitochondrial resilience, energetic flexibility	Tests whether dapagliflozin modifies brain metabolism *in vivo*
Functional/Cognitive phenotype	Standardized neuropsychological composites; executive function; memory measures	Anchors mechanistic biomarkers to clinically interpretable change	Distinguishes biological signal from meaningful functional benefit

Abbreviations: BBB, blood-brain barrier; CSF, cerebrospinal fluid; eGFR, estimated glomerular filtration rate; FDG-PET, fluorodeoxyglucose positron emission tomography; GFAP, glial fibrillary acidic protein; HOMA-IR, homeostatic model assessment of insulin resistance; NAA, N-acetylaspartate; NfL, neurofilament light chain; NT-proBNP, N-terminal pro-B-type natriuretic peptide. The proposed roadmap favors multicenter, double-blind, biomarker-enriched Phase IIb, trials of 18–36 months in metabolically vulnerable populations.

## 10 Integration with the wider therapeutic landscape

Dapagliflozin should not be viewed in isolation from the broader therapeutic landscape of cognitive impairment. Current and emerging strategies include anti-amyloid approaches (Frisoni et al., 2022), vascular risk modification, anti-inflammatory interventions, and multidomain lifestyle-based prevention frameworks (Livingston et al., 2024; Heneka et al., 2015; Jack et al., 2018). The unique potential of dapagliflozin lies in its ability to target upstream cardiometabolic and hemodynamic pathways that are often underrepresented in traditional neurodegenerative models of treatment (Ferrannini, 2017; Wiviott et al., 2019; McMurray et al., 2019; Heerspink et al., 2020; Vallon and Thomson, 2017).

This positioning suggests that dapagliflozin may ultimately prove most valuable not as a stand-alone cognitive therapy but as part of a broader risk-modifying strategy in selected patients (Livingston et al., 2024; Wiviott et al., 2019; McMurray et al., 2019; Heerspink et al., 2020). In metabolically vulnerable populations, combining systemic disease modification with biomarker-guided neurological assessment may offer a more realistic translational path than attempting to treat cognitive impairment through narrowly CNS-focused mechanisms alone (Jack et al., 2018; Palmqvist et al., 2020; Hansson, 2021; Zlokovic, 2011).

## 11 Discussion

Dapagliflozin should currently be regarded as a mechanistically plausible metabolic-neurovascular intervention for cognitive impairment rather than a proven cognition-directed therapy. The available evidence supports a biologically coherent rationale linking renal SGLT2 inhibition to systemic changes that may be relevant to brain health, including improved glycemic control, reduced blood pressure burden, attenuation of oxidative and inflammatory stress, favorable cardiorenal hemodynamics, and shifts in whole-body energy metabolism (Ferrannini, 2017; Wiviott et al., 2019; McMurray et al., 2019; Heerspink et al., 2020; Mosconi, 2005; Vallon and Thomson, 2017). When considered together, these effects provide a credible pharmacological basis for hypothesizing downstream benefits at the level of the neurovascular unit, mitochondrial function, glial activation, and synaptic resilience (Wardlaw et al., 2019; Sweeney et al., 2018; Heneka et al., 2015; Swerdlow, 2018; Zlokovic, 2011). However, the strength of evidence is not uniform across these domains, and the distinction between established systemic pharmacology and putative brain-directed effects remains central to any balanced interpretation.

A major strength of the current field is that preclinical studies have identified several convergent mechanisms through which dapagliflozin may influence pathways relevant to cognitive decline. Across experimental models, reported effects include reductions in oxidative stress and neuroinflammatory signaling, modulation of AMPK-mTOR and autophagy-related pathways, improvement of mitochondrial homeostasis, and changes in signaling networks implicated in synaptic plasticity, including Wnt/β-catenin and CREB/BDNF-associated mechanisms (El-Safty et al., 2022; Samman et al., 2023; Kostrzewska et al., 2025). These findings support the view that dapagliflozin may influence cognitive vulnerability through more than glucose lowering alone. At the same time, these studies must be interpreted cautiously, as many currently used models incompletely reflect the complexity of human cognitive impairment, particularly the coexistence of aging, insulin resistance, vascular injury, kidney dysfunction, and mixed neurodegenerative pathology (El-Safty et al., 2022; Samman et al., 2023).

The human evidence is substantially less definitive than the experimental literature. Observational studies suggesting lower dementia incidence among users of SGLT2 inhibitors are clinically provocative, but they do not establish causality and remain susceptible to residual confounding, treatment-selection bias, healthy-user effects, and competing-risk distortions (Pan et al., 2025; Shin et al., 2024). Randomized clinical trials have also not yet provided convincing proof of a cognition-specific benefit, largely because most were designed for glycemic, cardiovascular, or renal endpoints rather than for neurocognitive outcomes (Burns et al., 2025; Seminer et al., 2025). Follow-up duration, population selection, endpoint sensitivity, and the absence of biomarker-informed stratification all limit the ability of existing trials to detect whether dapagliflozin meaningfully alters cognitive trajectories. Accordingly, any claim that dapagliflozin prevents dementia or slows established neurodegeneration would currently exceed the available evidence.

One of the key unresolved issues is whether the potential cognitive relevance of dapagliflozin depends primarily on indirect systemic mechanisms or on meaningful direct effects within the central nervous system. From a translational pharmacology perspective, this distinction matters greatly. If benefit is largely mediated through peripheral unloading of metabolic and vascular stress, then the drug may be most relevant in individuals whose cognitive vulnerability is tightly linked to diabetes, obesity, chronic kidney disease, heart failure, endothelial dysfunction, or cerebral small-vessel disease (Livingston et al., 2024; Biessels and Despa, 2018; Wiviott et al., 2019; McMurray et al., 2019; Heerspink et al., 2020; van Sloten et al., 2020). If, by contrast, clinically important benefit requires direct central target engagement, then the current evidence base remains insufficient, as the extent of brain penetration and the functional relevance of central SGLT2-related pathways in humans are not yet clearly established (Kostrzewska et al., 2025; Vallon and Thomson, 2017). Future work should therefore avoid collapsing these mechanistic possibilities into a single narrative and instead explicitly test them using pharmacokinetic, imaging, and biomarker-based approaches (Jack et al., 2018; Palmqvist et al., 2020; Hansson, 2021).

These uncertainties also have important implications for trial design. The most informative future studies are unlikely to be broad, unselected dementia-prevention trials. Rather, they should enrich for metabolically vulnerable phenotypes in whom dapagliflozin’s known pharmacology is biologically most likely to intersect with mechanisms of brain injury (Wiviott et al., 2019; McMurray et al., 2019; Heerspink et al., 2020; Wardlaw et al., 2019; van Sloten et al., 2020; Zlokovic, 2011). Such populations may include individuals with type 2 diabetes and early cognitive decline, patients with chronic kidney disease or heart failure and evidence of cerebral small-vessel disease, or those with multimorbid metabolic-vascular risk profiles accompanied by inflammatory or neurodegenerative biomarker abnormalities (Livingston et al., 2024; Biessels and Despa, 2018; Wardlaw et al., 2019; van Sloten et al., 2020; Jack et al., 2018). In these settings, mechanistic endpoints should be integrated prospectively, including measures of insulin resistance, endothelial function, neuroimaging of perfusion and structural injury, and fluid biomarkers reflecting neurodegeneration, astroglial activation, and axonal injury (Jack et al., 2018; Palmqvist et al., 2020; Hansson, 2021; Zlokovic, 2011). Without this level of phenotypic and mechanistic precision, clinically meaningful effects may remain obscured by biological heterogeneity.

Taken together, the current literature supports cautious optimism but not therapeutic certainty. Dapagliflozin is best understood as a candidate modulator of interconnected metabolic, vascular, inflammatory, and cellular stress pathways that may shape brain resilience in selected populations (Ferrannini, 2017; Wiviott et al., 2019; McMurray et al., 2019; Heerspink et al., 2020; El-Safty et al., 2022; Samman et al., 2023; Kostrzewska et al., 2025; Vallon and Thomson, 2017). Its strongest present value lies in offering a pharmacologically grounded framework for studying how systemic disease modification might alter risk architecture for cognitive impairment (Livingston et al., 2024; Biessels and Despa, 2018; van Sloten et al., 2020; Craft, 2009). The field should now move beyond broad association and toward explicitly mechanism-linked translational studies that can determine whether dapagliflozin has measurable CNS-relevant effects, identify the patient groups most likely to benefit, and define whether observed cognitive signals reflect symptomatic change, slowed injury, or both (Burns et al., 2025; Seminer et al., 2025; Jack et al., 2018; Palmqvist et al., 2020; Hansson, 2021).

## 12 Conclusion

The investigation of dapagliflozin for cognitive impairment represents a critical intersection of metabolic pharmacology and neurobiology. A synthesis of current evidence reveals a distinct translational dichotomy: while preclinical models consistently demonstrate robust neuroprotective effects mediated by improved systemic metabolism, reduced inflammation, and preserved neurovascular integrity, human clinical data remain ambiguous. Large-scale observational studies strongly suggest a class-level reduction in dementia incidence among patients with type 2 diabetes, yet early randomized controlled trials have not yet confirmed specific cognitive or biomarker benefits.

The central unresolved question in the field is whether the cognitive benefits of dapagliflozin rely entirely on the attenuation of peripheral cardiometabolic stress, the “systemic-vascular corridor,” or whether they also involve direct target engagement within the central nervous system. Resolving this uncertainty requires a fundamental shift in trial design. Rather than pursuing broad, unselected dementia-prevention trials, future research must adopt a precision-pharmacology approach. By utilizing biomarker-enriched cohorts, specifically targeting patients in whom metabolic dysfunction, vascular brain injury, and early cognitive decline tightly co-segregate, investigators can isolate the specific phenotypes most likely to benefit. Ultimately, dapagliflozin is best positioned not as a standalone dementia treatment, but as a mechanistically targeted intervention to mitigate the profound metabolic and vascular drivers of cognitive decline.

### 12.1 Review methodology

This narrative review summarizes experimental, translational, and clinical literature relevant to dapagliflozin and cognitive impairment, with emphasis on pharmacological mechanisms, neurovascular biology, and cognition-related outcomes. Literature was identified from major biomedical databases (PubMed, Scopus, and Web of Science) using combinations of terms related to dapagliflozin, SGLT2 inhibition, cognition, dementia, Alzheimer’s disease, neuroinflammation, mitochondrial dysfunction, vascular brain injury, and neurovascular unit biology. The search window covered publications up to the time of manuscript preparation, with priority given to studies providing mechanistic insight, translational relevance, or synthesis of human evidence. Observational, randomized, and preclinical findings were evaluated separately to avoid conflation of evidentiary strength. This review is not intended as a formal systematic review or meta-analysis.
